# Comparison of biparametric and multiparametric MRI in the diagnosis of prostate cancer

**DOI:** 10.1186/s40644-019-0274-9

**Published:** 2019-12-21

**Authors:** Lili Xu, Gumuyang Zhang, Bing Shi, Yanhan Liu, Tingting Zou, Weigang Yan, Yu Xiao, Huadan Xue, Feng Feng, Jing Lei, Zhengyu Jin, Hao Sun

**Affiliations:** 1 Department of Radiology, Peking Union Medical College Hospital, Peking Union Medical College, Chinese Academy of Medical Sciences, Shuaifuyuan No.1, Wangfujing Street, Dongcheng District, Beijing, 100730 China; 2 Department of Urology, Peking Union Medical College Hospital, Peking Union Medical College, Chinese Academy of Medical Sciences, Beijing, 100730 China; 3 Department of Pathology, Peking Union Medical College Hospital, Peking Union Medical College, Chinese Academy of Medical Sciences, Beijing, 100730 China

**Keywords:** Prostate cancer, Magnetic resonance imaging, Dynamic contrasted-enhanced imaging, Prostate imaging reporting and data system

## Abstract

**Purpose:**

To compare the diagnostic accuracy of biparametric MRI (bpMRI) and multiparametric MRI (mpMRI) for prostate cancer (PCa) and clinically significant prostate cancer (csPCa) and to explore the application value of dynamic contrast-enhanced (DCE) MRI in prostate imaging.

**Methods and materials:**

This study retrospectively enrolled 235 patients with suspected PCa in our hospital from January 2016 to December 2017, and all lesions were histopathologically confirmed. The lesions were scored according to the Prostate Imaging Reporting and Data System version 2 (PI-RADS V2). The bpMRI (T2-weighted imaging [T2WI], diffusion-weighted imaging [DWI]/apparent diffusion coefficient [ADC]) and mpMRI (T2WI, DWI/ADC and DCE) scores were recorded to plot the receiver operating characteristic (ROC) curves. The area under the curve (AUC), accuracy, sensitivity, specificity, negative predictive value (NPV), and positive predictive value (PPV) for each method were calculated and compared. The patients were further stratified according to bpMRI scores (bpMRI ≥3, and bpMRI = 3, 4, 5) to analyse the difference in DCE MRI between PCa and non-PCa lesions (as well as between csPCa and non-csPCa).

**Results:**

The AUC values for the bpMRI and mpMRI protocols for PCa were comparable (0.790 [0.732–0.840] and 0.791 [0.733–0.841], respectively). The accuracy, sensitivity, specificity, PPV and NPV of bpMRI for PCa were 76.2, 79.5, 72.6, 75.8, and 76.6%, respectively, and the values for mpMRI were 77.4, 84.4, 69.9, 75.2, and 80.6%, respectively. The AUC values for the bpMRI and mpMRI protocols for the diagnosis of csPCa were similar (0.781 [0.722–0.832] and 0.779 [0.721–0.831], respectively). The accuracy, sensitivity, specificity, PPV and NPV of bpMRI for csPCa were 74.0, 83.8, 66.9, 64.8, and 85.0%, respectively; and 73.6, 87.9, 63.2, 63.2, and 87.8%, respectively, for mpMRI. For patients with bpMRI scores ≥3, positive DCE results were more common in PCa and csPCa lesions (both *P* = 0.001). Further stratification analysis showed that for patients with a bpMRI score = 4, PCa and csPCa lesions were more likely to have positive DCE results (*P* = 0.003 and *P* < 0.001, respectively).

**Conclusion:**

The diagnostic accuracy of bpMRI is comparable with that of mpMRI in the detection of PCa and the identification of csPCa. DCE MRI is helpful in further identifying PCa and csPCa lesions in patients with bpMRI ≥3, especially bpMRI = 4, which may be conducive to achieving a more accurate PCa risk stratification. Rather than omitting DCE, we think further comprehensive studies are required for prostate MRI.

## Introduction

Prostate cancer (PCa) is the most frequently diagnosed cancer among men in over one-half of the countries of the world [1], and it is also the second leading cause of cancer-related death in the United States [2]. The age-adjusted incidence rates of PCa in developing Western countries and Asia has increased progressively with time [3, 4]. The early and accurately diagnosis of PCa and identification of clinically significant PCa (csPCa), which requires more positive treatment, for reducing mortality due to aggressive PCa and avoid unnecessary treatment remains challenging in clinical practice.

Multiparametric magnetic resonance imaging (mpMRI) has emerged as an important tool in the early diagnosis of PCa and is particularly helpful in the detection, local staging, and estimation of the aggressiveness of prostate cancer lesions [5, 6]. mpMRI is currently recognized as the best imaging method for assessing prostate cancer [7, 8]. According to the recommendations of the Prostate Imaging Reporting and Data System version 2 (PI-RADS V2), mpMRI includes T1- and T2-weighted imaging (T2WI), diffusion-weighted imaging (DWI) and dynamic contrast-enhanced (DCE) MRI [9]. However, the value of DCE MRI in the detection of prostate cancer is still controversial. Some studies have shown that combining DCE MRI with T2WI and DWI does not significantly improve the diagnostic accuracy of prostate cancer [10–12]. Because DCE MRI is a time-consuming process with additional costs and a potential risk of nephrogenic systemic fibrosis, a biparametric MRI (bpMRI) protocol has been proposed. This imaging protocol omits the DCE MRI and only evaluates the T2WI and DWI sequences of the prostate [10, 13, 14]. Nevertheless, some studies have found that DCE MRI is highly sensitive in the diagnosis of PCa [15, 16], especially in peripheral lesions, and combining DCE MRI with DWI can significantly improve the accuracy of cancer detection [17]. Presently, in PI-RADS V2, DCE MRI can be used to upgrade lesions graded as PI-RADS 3 to PI-RADS 4 in the peripheral zone.

This study was designed to compare the diagnostic performance of bpMRI and mpMRI for PCa and csPCa, and to further explore the added value of DCE MRI in combination with T2WI and DWI in the evaluation of prostate lesions.

## Methods

### Patients

Patients who underwent prostate mpMRI for suspected prostate cancer at Peking Union Medical College Hospital from January 2016 to December 2017 were retrospectively enrolled in this study. Ethics approval was obtained and informed consent was waived for this study. Inclusion criteria: (1) standardized prostate mpMRI performed for all patients; (2) standardized prostatic biopsy and/or prostatectomy performed after MRI examination and their pathological results made available; and (3) no radiation therapy, hormonal therapy or other treatments prior to MRI examination. Exclusion criteria: (1) unsatisfactory quality of MRI images; (2) biopsy or other therapies performed before MRI examination; and (3) pathological results unavailable. This study finally enrolled 235 patients; 180 underwent biopsy, and 55 underwent prostatectomy.

### MRI protocol

A 3.0 T MRI scan system (GE750, GE Healthcare) with an abdominal eight-channel surface phased array coil was used to perform the imaging. The pulse sequences and MR imaging acquisition parameters applied in this study are shown in Table 1. Transverse, sagittal, and frontal T2WI images, DWI images with multiple b values and corresponding ADC maps were obtained for analysis. DCE images were obtained after intravenous injection of gadopentetate dimeglumine (Magnevist; Bayer Healthcare) at a dose of 0.1 mmol/kg of body weight and a rate of 3 mL/sec by using an automatic injector (Spectris Solaris EP; Medrad).

**Table 1:** Sequence parameters for prostate multiparametric MRI

Parameters	T2WI	DWI	DCE
Sequence	FRFSE	SE-EPI	3D-GRE
TR/TE (ms)	4137/86	4200/90	4.3/1.3
Flip angle (degree)	110	90	12
Echo train length	32	1	N/A
Field of view (mm × mm)	270 × 270	360 × 360	400 × 400
Matrix size	288 × 192	128 × 96	320 × 192
Thickness (mm)	3.0	3.0	3.0
Other		b values = 100, 150, 200, 500, 800, 1000, 1500, 2000 mm^2^/sec	Temporal resolution <10s, total scan time of 5 min

*TR* Repetition time, *TE* Time echo, *FRFSE* Fast relaxation fast spin echo, *SE-EPI* Spin-echo echo planar imaging, *3D-GRE* 3D-gradient echo

### Image analysis

**Fig. 1:**
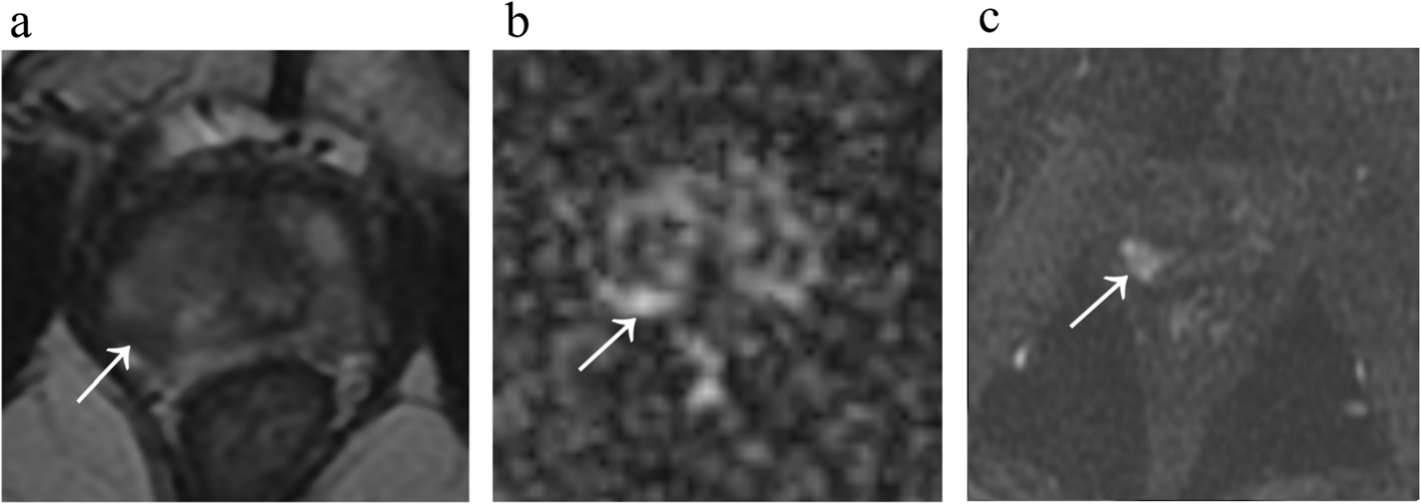
Images from a 73-year-old man with a PSA level of 8.4 ng/mL. **a** A focal hypointensity with a partially circumscribed margin is shown in the right posterior peripheral zone on axial T2-weighted MRI, with a T2WI score of 4. **b** DWI shows slightly increased signal intensity of the lesion with a score of 3. **c** DCE MRI reveals the lesion with an early and clear enhancement, which translates to a positive DCE score. The PI-RADS category of this lesion is 3 with the bpMRI protocol and 4 with the mpMRI protocol. The lesion was proven to be a clinically significant cancer with a Gleason Score = 4 + 3 by biopsy

Two radiologists experienced in interpreting prostate MRI (5 and 13 years) who were blinded to the pathological results reviewed the MRI images according to PI-RADS V2 and recorded the scores from the T2WI, DWI and DCE MRI for each lesion (Fig. 1). First, the radiologists analysed T2WI and DWI images and ADC maps for each patient and recorded the T2WI score and DWI/ADC score separately. For transition zone lesions, the combined T2WI and DWI/ADC score was sufficient for categorization. For peripheral zone lesions, as suggested by the PI-RADS for cases “without adequate DCE”, the categories were determined by DWI assessment alone. Subsequently, DCE images were supplied to the radiologists for assessment without a rest period. Peripheral lesions with bpMRI PI-RADS category 3 were then upgraded to PI-RADS category 4 when early enhancement was observed on DCE MRI. For controversial cases, the two radiologists jointly negotiated and reached an agreement for both the bpMRI score and mpMRI score.

### Standard of references

The histological grading method for PCa was performed using the Gleason grading system [18]. As proposed in the PI-RADS V2, csPCa is defined as a tumor with a Gleason score ≥ 7 and/or a volume ≥ 0.5 cm^3^ and/or extra-prostatic invasion [9]. When multiple lesions were present, the lesion with the highest PI-RADS score was used in the statistical analysis. To match the lesions on MR images with histopathologic specimens from biopsy or prostatectomy, we first roughly located the highest PI-RADS category lesions on MR images to the corresponding 11-region map and then obtained the biopsy/prostatectomy results for the relevant regions.

### Statistical analysis

Using the pathological results as the standard of reference, the receiver operating characteristic (ROC) curves for bpMRI and mpMRI were plotted, and the diagnostic accuracy, sensitivity, specificity, positive predictive value (PPV) and negative predictive value (NPV) and their 95% confidence intervals (CI) were calculated to evaluate the diagnostic performance of the two scoring methods. A Delong test was used to assess the difference between areas under the curve (AUC). In detail, for the accuracy in detecting PCa or csPCa, PI-RADS V2 category 1–3 were considered negative, while PI-RADS V2 category 4–5 were considered positive. Patients with bpMRI PI-RADS ≥3 were further stratified according to bpMRI category to assess the difference in DCE MRI between PCa and non-PCa lesions (as well as between csPCa and non-csPCa). The chi-square test or Fisher’s exact test was used to test for differences in dichotomized score values. The difference was considered statistically significant with a two-sided *P* value < 0.05. Statistical analysis was performed using SPSS 22.0 (IBM) and MedCal 15.0 software (MedCalc Software).

## Results

### Clinicopathological data

**Fig. 2:**
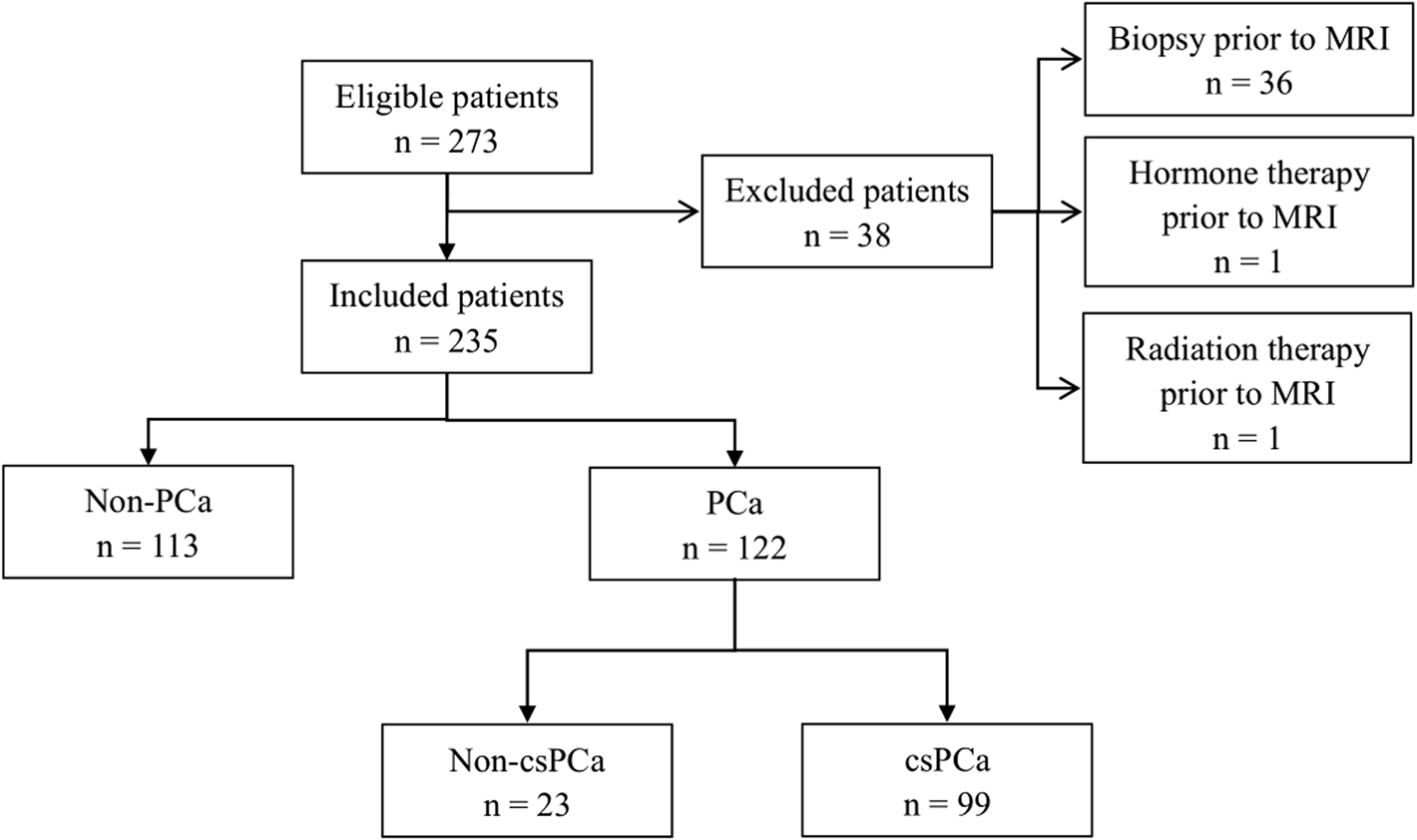
Flow diagram of the inclusion and exclusion criteria in this research

Figure 2 shows a flowchart of patient recruitment. With a mean age of 66.87 ± 8.53 years and a median PSA level of 4.65 (0.22–86.00) ng/ml, a total of 235 patients who met the criteria mentioned above were enrolled in this study. Histopathological analysis revealed PCa in 122 (51.9%) of these patients and non-PCa in 113 (48.1%). Among the 122 patients with PCa, 99 were defined as csPCa (67 with Gleason score = 3 + 4, and 32 with Gleason score ≥ 4 + 4) (Table 2).

**Table 2:** Clinicopathological data of patients included in this study

Clinicopathological data	All patients (*n* = 235)
Age (year), mean ± SD	66.87 ± 8.53
PSA (ng/mL), median (range)	4.65 (0.22–86.00)
PCa, n (%)	122 (51.9)
csPCa, n (%)	99 (42.1)
Gleason score, n (%)	
3 + 3	23 (9.8)
3 + 4	42 (17.8)
4 + 3	25 (10.6)
3 + 5	1 (0.4)
4 + 4	9 (3.8)
4 + 5	15 (6.4)
5 + 4	5 (2.1)
5 + 5	2 (0.9)

### Comparison of the diagnostic performances of bpMRI and mpMRI

**Fig. 3:**
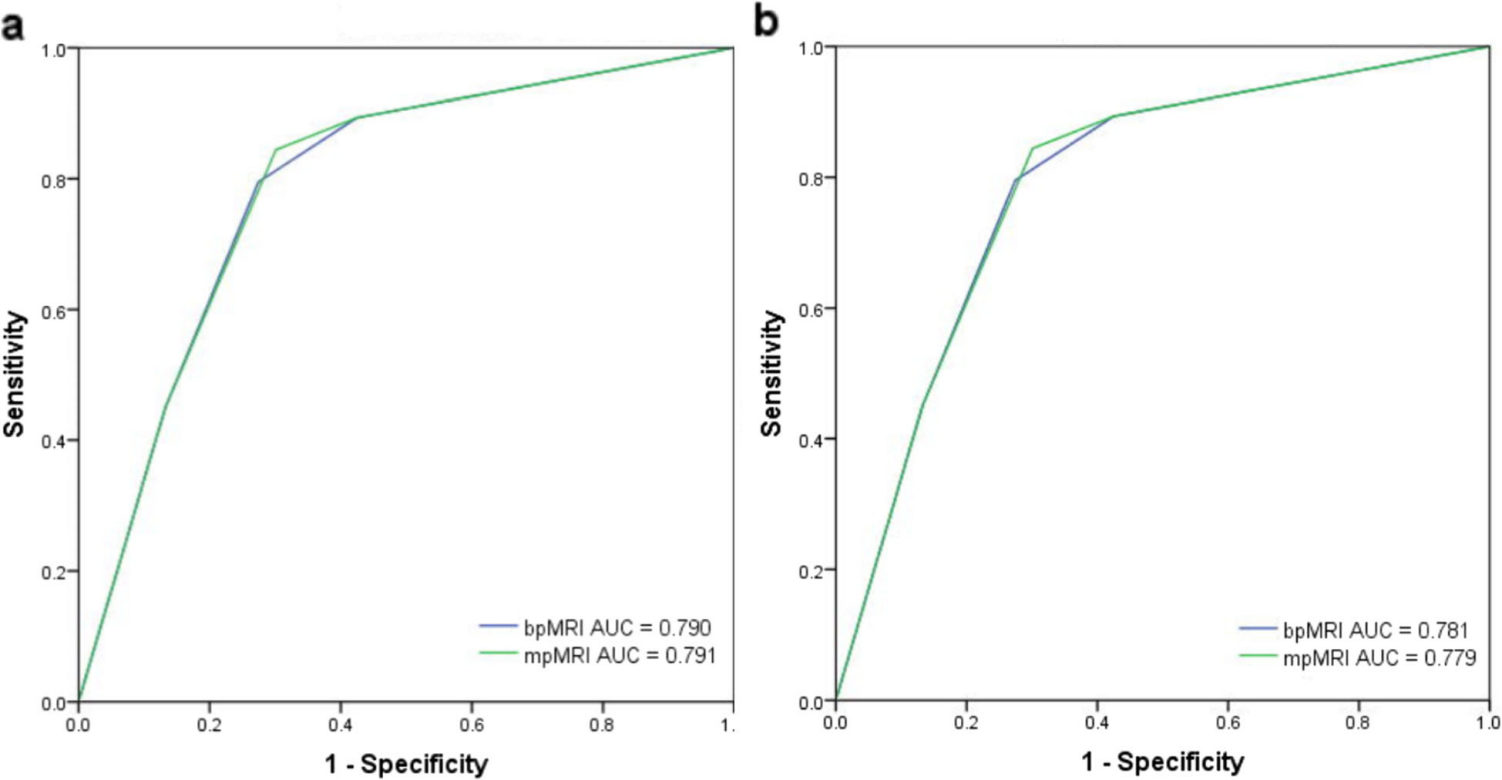
Comparison of ROC curves between bpMRI and mpMRI for prostate cancer (**a**) and clinically significant cancer detection (**b**)

The AUCs for the bpMRI and mpMRI protocols in diagnosing PCa were 0.790 (0.732–0.840) and 0.791 (0.733–0.841), respectively, and the difference was not statistically significant (*P* > 0.05). The accuracy, sensitivity, specificity, PPV and NPV of bpMRI for diagnosing PCa were 76.2, 79.5, 72.6, 75.8, and 76.6%, respectively; the values for mpMRI were 77.4, 84.4, 69.9, 75.2, and 80.6%, respectively. For the diagnosis of csPCa, the AUC values of bpMRI and mpMRI were 0.781 (0.722–0.832) and 0.779 (0.721–0.831), respectively, which were not significantly different (*P* > 0.05). The accuracy, sensitivity, specificity, PPV and NPV of bpMRI were 74.0, 83.8, 66.9, 64.8, and 85.0%, respectively, and the values for mpMRI were 73.6, 87.9, 63.2, 63.2, and 87.8%, respectively (Table 3 and Fig. 3).

**Table 3:** Comparison of mpMRI and bpMRI for diagnosing prostate lesions

	mpMRI		bpMRI		*P*
	< 4 (*n* = 98)	≥ 4 (*n* = 137)	< 4 (*n* = 107)	≥ 4 (*n* = 128)	
Non-PCa, n (%)	79 (80.6)	34 (24.8)	82 (76.6)	31 (24.2)	
PCa, n (%)	19 (19.4)	103 (75.2)	25 (23.4)	97 (75.8)	
Accuracy		77.4 (71.7–82.3)		76.2 (70.3–81.2)	
Sensitivity		84.4 (76.8–90.4)		79.5 (71.3–86.3)	
Specificity		69.9 (60.6–78.2)		72.6 (63.4–80.5)	
PPV		75.2 (67.1–82.2)		75.8 (67.4–82.9)	
NPV		80.6 (71.3–87.9)		76.6 (67.5–84.3)	
AUC		0.791 (0.733–0.841)		0.790 (0.732–0.840)	0.760^*^
Non-csPCa, n (%)	86 (87.8)	50 (36.5)	91 (85.0)	45 (35.2)	
csPCa, n (%)	12 (12.2)	87 (63.5)	16 (15.0)	83 (64.8)	
Accuracy		73.6 (67.6–78.9)		74.0 (68.1–79.2)	
Sensitivity		87.9 (79.8–93.6)		83.8 (75.1–90.5)	
Specificity		63.2 (54.5–71.3)		66.9 (58.3–74.7)	
PPV		63.2 (54.9–71.6)		64.8 (55.9–73.1)	
NPV		87.8 (79.6–93.5)		85.0 (76.8–91.2)	
AUC		0.779 (0.721–0.831)		0.781 (0.722–0.832)	0.753^*^

The accuracy, sensitivity, specificity, positive predictive value (PPV) and negative predictive value (NPV) are presented as (%, [95% confidence interval]); the areas under the curve (AUCs) are presented with 95% confidence intervals

^*^Delong test was used to compare the AUCs of bpMRI and mpMRI

### DCE score for the diagnosis of PCa and csPCa

For patients with bpMRI scores ≥3, the DCE score was statistically significant between PCa and non-PCa and between csPCa and non-csPCa patients (both *P* = 0.001). That is, DCE-positive results were more common in PCa and csPCa lesions among these patients. After stratifying according to bpMRI score, the difference in DCE between PCa and non-PCa and between csPCa and non-csPCa was statistically significant in patients with bpMRI score = 4 (*P* = 0.003, and *P* < 0.001, respectively), which means that among these patients, PCa and csPCa lesions were more likely to have early enhancement in DCE MRI. However, for patients with bpMRI = 3 and bpMRI = 5, no significant difference was noted (all *P* > 0.05) (Table 4).

**Table 4:** The differences in DCE in patients with bpMRI ≥3 (n, %)

	Non-PCa (*n* = 48)	PCa (*n* = 109)	P^*^	Non-csPCa (*n* = 66)	csPCa (*n* = 91)	P^*^
bpMRI ≥3 (*n* = 157)			0.001			0.001
DCE (−)	19 (39.6)	17 (15.6)		24 (36.4)	12 (13.2)	
DCE (+)	29 (60.4)	92 (84.4)		42 (63.6)	79 (86.8)	
bpMRI = 3 (*n* = 29)			0.286			0.943
DCE (−)	9 (52.9)	4 (33.3)		10 (47.6)	3 (37.5)	
DCE (+)	8 (47.1)	8 (66.7)		11 (52.4)	5 (62.5)	
bpMRI = 4 (*n* = 58)			0.003			< 0.001
DCE (−)	9 (56.3)	6 (14.3)		12 (52.2)	3 (8.6)	
DCE (+)	7 (43.8)	36 (85.7)		11 (47.8)	32 (91.4)	
bpMRI = 5 (*n* = 70)			0.844			0.991
DCE (−)	1 (6.7)	7 (12.7)		2 (9.1)	6 (12.5)	
DCE (+)	14 (93.3)	48 (87.3)		20 (90.9)	42 (87.5)	

^*^Compared by chi-square test or Fisher’s exact test

## Discussion

The results of this study indicate that the bpMRI protocol is comparable to the mpMRI protocol in the diagnosis of PCa and csPCa. For patients with a bpMRI score ≥ 3, the DCE score was significantly different between PCa and non-PCa and between csPCa and non-csPCa. Further stratification analysis showed that in patients with a bpMRI score = 4, a significant difference was also noted in the DCE score. The results indicated that PCa and csPCa lesions were more likely to have positive DCE results in bpMRI ≥3 patients (especially bpMRI = 4 patients), suggesting that for these patients, the application of DCE MRI may help to improve the tumor detection rate and achieve a more accurate tumor aggressiveness classification.

Currently, mpMRI is the standard method for prostate imaging and plays an important role in the detection, targeted biopsy, local staging and risk classification of prostate cancers. PI-RADS has been proposed to standardize the scan process, image interpretation and report writing of prostate mpMRI, to improve the management of prostate cancer patients [9]. Compared with the PI-RADS proposed in 2012, the main change in PI-RADS V2 is the proposal of a dominant sequence, in which T2WI is proposed as the dominant sequence for transition zone lesions and DWI for peripheral zone lesions. DCE MRI plays a supplementary role only when DWI is not sufficient for diagnosis [19, 20], and DCE positivity only upgrades DWI category 3 lesions in the peripheral zone to category 4.

Considering the long acquisition time of DCE MRI, its potential risks and additional costs, patient discomfort associated with contrast agent injection, and the relatively small incremental benefit of DCE MRI, several authors have suggested that it be abandoned [21, 22]. Some studies have compared the diagnostic performance of bpMRI and mpMRI [13, 23]. The research of Junker et al. [23] showed that bpMRI and mpMRI have comparable diagnostic performance in detecting prostate cancer (AUC = 0.914 and 0.917, respectively). Their comparability was also demonstrated by some meta-analyses published recently [24–28]. From a head-to-head comparison in detecting PCa, mpMRI had a significantly higher pooled sensitivity than bpMRI [25]. Kuhl et al. [13] investigated 542 patients with PSA ≥ 3 ng/mL and negative transrectal ultrasonography-guided biopsy findings, and found that bpMRI and mpMRI have similar accuracy in diagnosing clinically significant cancer (89.1% vs. 87.2%); between-reader agreement for the bpMR protocol was substantial (k = 0.81), while the agreement for mpMRI was moderate (k = 0.60). Our results also indicated the comparability of bpMRI and mpMRI in the detection of prostate cancer and the identification of clinically significant lesions.

Whether DCE imaging has added benefits in the diagnosis and risk stratification of prostate cancer has also been widely studied. Greer et al. [17] reported that in the peripheral zone, DCE positivity improved the probability of cancer detection of PI-RADS category 2, 3, and 4 lesions (OR, 2.00; *P* = 0.027); for these lesions, the tumor detection rate increased by 15.7, 16.0, and 9.2%, respectively. These results suggest that the DCE sequence has important application value in the diagnosis of prostate cancer in the peripheral zone, which may help improve the risk stratification accuracy of prostate cancer. However, since the number of transition zone lesions was limited in this study, DCE was not found to significantly increase the tumor detection rate in the transition zone. The study by Rosenkrantz et al. [29] analysed the role of the DCE sequence in the transition zone. This study selected 106 patients with suspected PCa, and three radiologists scored the transition zone using the pathological results from prostatectomy as a reference. The results showed that scoring according to T2WI + DWI had higher sensitivity than depending on T2WI alone, but the addition of DCE did not improve the diagnostic sensitivity, so DCE was considered useless in the analysis of transition zone lesions. Our study showed that, for patients with bpMRI category ≥3, DCE was noted to be statistically significant in the diagnosis of prostate cancer and the identification of clinically significant disease. Further stratification analysis showed a significant difference in DCE between PCa and non-PCa and between csPCa and non-csPCa among patients with bpMRI category = 4, which indicates that for those patients, DCE may help improve the tumor detection rate and the accuracy of tumor aggressiveness classification. The research of Rosenkrantz et al. [30] proposed some adjustments to the decision rules of PI-RADS and found that for transition zone lesions upgraded from category 3 to 4 based on a DCE score of positive when integrating new criteria (unencapsulated sheet-like enhancement), the frequency of Gleason score ≥ 7 tumors was 33.3–57.1%. Rather than omitting DCE, further comprehensive studies are required for future updates of the existing system.

There are some limitations in this study. First, the sample size of our study is not very large. Second, due to the limited sample size, we did not separate lesions into peripheral and transition zones and investigate the added value of DCE in different zones. Third, most patients had theire biopsy results used as the standard of reference, which may have underestimated the diagnostic performance of mpMRI and bpMRI due to the high false negative rate of biopsy [31]. Further studies including more patients are ongoing to verify the conclusion of this study and analyse the added value of DCE MRI in different zones. In addition to providing qualitative parameters, DCE MRI can also provide a wealth of quantitative and semi-quantitative parameters [32]. Whether these other parameters are useful in the early diagnosis of prostate cancer also needs to be tested.

## Conclusion

In conclusion, the results of this study indicated that bpMRI is comparable to mpMRI in the diagnosis of PCa and csPCa, but DCE MRI is helpful in further identifying PCa and csPCa in patients with bpMRI ≥3, especially bpMRI = 4, which may help achieve a more accurate aggressiveness classification and individualized treatment of prostate cancer.

## Data Availability

The datasets used and/or analysed during the current study are available from the corresponding author on reasonable request.

## Abbreviations

AUC: Area under the curve
bp: Biparametric
csPCa: Clinically significant prostate cancer
DCE: Dynamic-contrast enhanced
DWI: Diffusion-weighted imaging
mp: Multiparametric
NPV: Negative predictive value
PCa: Prostate cancer
PI-RADS: Prostate Imaging Reporting and Data System
PPV: Positive predictive value
PSA: Prostate specific antigen
ROC: Receiver operating characteristic
T2WI: T2-weighted imaging
